# Reducing inappropriate infectious disease serology tests through the use of decision support in a provincial electronic health system

**DOI:** 10.1128/jcm.00770-26

**Published:** 2026-09-25

**Authors:** R. Benson Weyant, Nathan J. Zelyas, Carmen L. Charlton, William Stokes, Hong Yuan Zhou, Sean Jiang, Maria Falsetti, Byron M. Berenger

**Affiliations:** 1 Division of Infectious Diseases, Cumming School of Medicine, University of Calgary 2129, Calgary, Alberta, Canada; 2 Provincial Laboratory for Public Health, Alberta Precision Laboratories, Edmonton, Alberta, Canada; 3 Department of Laboratory Medicine and Pathology, University of Alberta 3158, Edmonton, Alberta, Canada; 4 Microbiology, Medical Microbiology, Canadian Blood Services, Edmonton, Alberta, Canada; 5 Surveillance and Discovery Laboratory, Canadian Blood Services 27365, Ottawa, Canada; 6 Provincial Laboratory for Public Health, Alberta Precision Laboratories 590576, Calgary, Alberta, Canada; 7 Department of Pathology & Laboratory Medicine, Cumming School of Medicine, University of Calgary 2129, Calgary, Alberta, Canada; 8 Provincial Support Services, Information Technology, Alberta Health Services 3146, Edmonton, Alberta, Canada; Mayo Clinic Minnesota, Rochester, Minnesota, USA

**Keywords:** laboratory utilization, efficiency, laboratory, diagnostic stewardship, electronic health records, laboratory information system, decision support systems, clinical, microbiological techniques, serology, carbon footprint

## Abstract

Diagnostic stewardship improves patient care and reduces health costs by promoting appropriate test use. Ordering serologic tests for infectious diseases can be challenging, with indications differing based on the clinical picture, previous results, or pathogens. We aimed to use our electronic health system (EHS) to provide real-time, patient-specific education on test appropriateness to reduce inappropriate testing. This observational study took place from October 2023 to 2024. The study included all users of the province-wide, integrated EHS in Alberta, Canada (population 4.9 million), which captures all laboratory orders across healthcare settings. Two diagnostic stewardship measures were assessed for 23 infectious disease serology tests: (i) Best Practice Advisories (BPAs) triggered at the point of ordering tests, and (ii) auto-cancellation rules that canceled the test upon receipt of the sample at the testing laboratory. BPAs flagged duplicate orders (within 3 days for IgM and 7 days for IgG), previously positive IgG, or missing prerequisite tests. Providers could override BPAs if necessary. After 1 year, BPAs led to 22,907 canceled tests (1.4% of targeted tests ordered). An additional 90,749 (5.3%) tests were auto-canceled upon laboratory receipt. Clinicians overrode BPAs on only 146 occasions (0.6%), with no signal of alert fatigue. BPAs prevented an estimated 16,744 phlebotomies and saved $52,049 CAD ($38,516 USD) in laboratory reagents. Auto-cancellation rules saved an additional $208,089 CAD ($153,986 USD). Integrating BPAs into an EHS effectively supports diagnostic stewardship at ordering by reducing unnecessary phlebotomies, improving test selection, and saving resources. When electronic ordering is unavailable, auto-cancellations upon laboratory receipt are an effective alternative.

**IMPORTANCE:**

Serology tests for past or current infections are among the most ordered laboratory investigations, yet a substantial proportion are unnecessary, particularly when patients have had recent testing or known immunity. Unneeded testing wastes resources, exposes patients to avoidable blood draws, and can produce misleading results that trigger further investigations. This study shows that two simple tools built into a province-wide electronic health record can meaningfully reduce inappropriate ordering of serologic tests across a wide range of viral and bacterial pathogens. Across nearly 1.7 million tests in 1 year, the interventions canceled more than 113,000 unnecessary tests, prevented thousands of blood draws, lowered carbon emissions, and saved roughly a quarter million Canadian dollars in laboratory reagents alone. As these tools require no additional personnel or infrastructure, the approach is widely applicable to clinical laboratories regardless of size or resource setting, supporting broader adoption of electronic decision support for efficient infection testing.

## INTRODUCTION

With an ever-increasing number of microbiology tests available, selecting the most appropriate test for each patient can be challenging (1). Ordering tests that are not indicated or inappropriate for the clinical scenario can lead to additional costs and unnecessary phlebotomy and increase the risk of both false-negative and false-positive results that may prompt further unnecessary investigations or treatments (2, 3). Diagnostic stewardship promotes the judicious use of diagnostic tests and aims to limit overuse to improve clinical outcomes and resource utilization (4).

Detection of pathogen-specific immunoglobulins or antigens is a cornerstone of infection diagnosis. Immunoglobulin M (IgM) typically indicates a recent or acute infection, whereas immunoglobulin G (IgG), which represents a previous infection, can remain detectable for decades or even lifelong, negating the need for repeated IgG testing. While IgG titers may rise with reactivation or reinfection with certain pathogens and some assays are used quantitatively or semi-quantitatively (3, 5), persistent IgG positivity is common following infection. Nevertheless, unnecessary repeat IgG testing occurs in up to 20% of cases (6, 7).

The advent of electronic health systems (EHS) has introduced a variety of tools to enhance diagnostic stewardship. These include best practice advisories (BPAs), clinical decision support (CDS) alerts, electronically assisted appropriateness tools, machine-guided decision rules, electronic look-back rules, automated alerts, and utilization management protocols (8). The implementation of EHS alerts has been found to decrease both financial costs and the environmental impact associated with laboratory testing (9–11). Our study aimed to leverage these EHS-based tools, particularly BPAs and automated order cancellation rules, to reduce unnecessary tests after clinicians receive real-time decision support at the point of ordering or after receipt of a sample at the laboratory.

## MATERIALS AND METHODS

### Study design

This observational study retrospectively evaluated the impact of BPAs and auto-cancellation rules on infectious disease serology test utilization post-implementation. The project was classified as a quality improvement initiative, and ethics review was deemed unnecessary by the Calgary Health Research Ethics Board.

### Study setting and participants

The study was conducted at clinical and laboratory sites across Alberta (population 4.9 million), utilizing the provincial EHS, which integrates both the electronic medical record (EMR) and laboratory information system (LIS). The EHS was rolled out in waves across the province from 2019 to 2024 and uses Epic Systems Software (Verona, WI). We assessed all infectious disease serology tests subject to auto-cancellation rules that were ordered or resulted through the EMR/LIS over a 1-year period from 1 October 2023 to 30 September 2024. The intervention applied to all clinicians and laboratory personnel involved in ordering or processing specific infectious disease serology tests within the EHS.

At the time of observation, both provincial laboratories for public health performed all target testing and used a shared LIS connected to the Epic EMR. Orders not generated directly within the EMR, including those printed from other EMRs or manually completed, were manually entered into the EHS at specimen collection or receiving sites so that all test requests and results were captured within the unified EMR/LIS environment. Starting in May 2023, acute care facilities, including outpatient clinics, urgent cares, and emergency departments, in the three most populous health zones placed orders using the Epic EMR. In November 2023, the Epic EMR expanded to all acute care facilities across all health zones in the province (12). Past serology results for all targeted tests on patients tested in Alberta since February 2014 are available in the EHS.

### Intervention

The intervention included the implementation of two complementary automated look-back rule-based diagnostic stewardship modalities: (i) BPAs at the point of ordering in the EMR (no blood drawn) and (ii) the auto-cancellation of tests at receipt in the laboratory (blood drawn and transported to the laboratory, but no test performed). These two modalities worked in tandem because BPAs only captured orders placed through the EMR. Orders entered via paper requisitions or phoned-in requests bypass the BPA entirely. Auto-cancellation served as a second checkpoint that applied the same criteria once a specimen physically arrived in the laboratory. The interventions were applied to 23 serologic tests (Table 1). Rule exceptions were made for specific populations (e.g., testing for organ donors) and for certain sexual assault screening orders (e.g., HBsAb) where prophylaxis may be indicated. Within sexual assault response team (SART) orders, this exception applied specifically to HBsAb, since waning immunity may warrant post-exposure prophylaxis regardless of a previous positive result. Standard historical-positivity and duplicate-order criteria continued to apply to syphilis, HIV, and HCV testing within the same panel.

**TABLE 1:** Targeted orders and associated BPA and auto-cancellation rules with suggested alternate tests if applicable^*b*^

Order	Repeat testing within	Previous result	Alternate test suggested
CMV IgG and IgM	3 days	Applies only to CMV IgM if previous positive/reactive IgG	None
HAV IgG	7 days	Positive/reactive IgG	None
HAV IgM	3 days	Positive/reactive IgG	None
HBcAb total (IgM + IgG)	7 days	Positive/reactive HBcAb total	None
HBcAb IgM	3 days	Positive/reactive HBcAb total^*a*^	None
HBsAb	7 days	Immune result ≥10 IU/L	None
HBsAg	7 days	No rule	None
HCV Ab	7 days	Confirmation positive (RNA or antibody)	HCV NAT
HIV-1/2 Ag/Ab	None	HIV-1/2 confirmation assay positive twice or once with positive HIV NAT or Western blot	HIV NAT
HTLV-1/2 Ab	7 days	HTLV confirmation positive	HTLV NAT
Measles IgG	7 days	Positive/reactive IgG	None
Mumps IgG	7 days	Positive/reactive IgG	None
Prenatal panel (HIV, rubella, VZV, and syphilis)	7 days	Individual components canceled as per component rules (auto-cancellation only)	None
Rapid HIV (lateral flow Ab)	7 days	HIV-1/2 confirmation assay positive twice or once with positive HIV NAT or Western blot	None
Rubella IgG	7 days	Immune result ≥10 IU/L	None
Syphilis EIA	7 days	INNO-LIA® or TPPA positive	RPR
VZV IgG	7 days	Positive/reactive IgG	None

^*a*^ Modified on 14 August 2024, to limit the rule to activate only if there was a previous positive/reactive result more than 6 months ago.

^*b*^ Ab, antibody; Ag, antigen; CMV, cytomegalovirus; HAV, hepatitis A virus; HBcAb, hepatitis B core antibody; HBQsAg, hepatitis B quantitative surface antigen; HBsAb, hepatitis B surface antibody; HBsAg, hepatitis B surface antigen; HCV, hepatitis C virus; HTLV, human T-lymphotropic virus; NAT, nucleic acid test; RPR, rapid plasma reagin; SART, sexual assault response team; TPPA, *Treponema pallidum* particle agglutination; VZV, varicella zoster virus.

**Fig 1:**
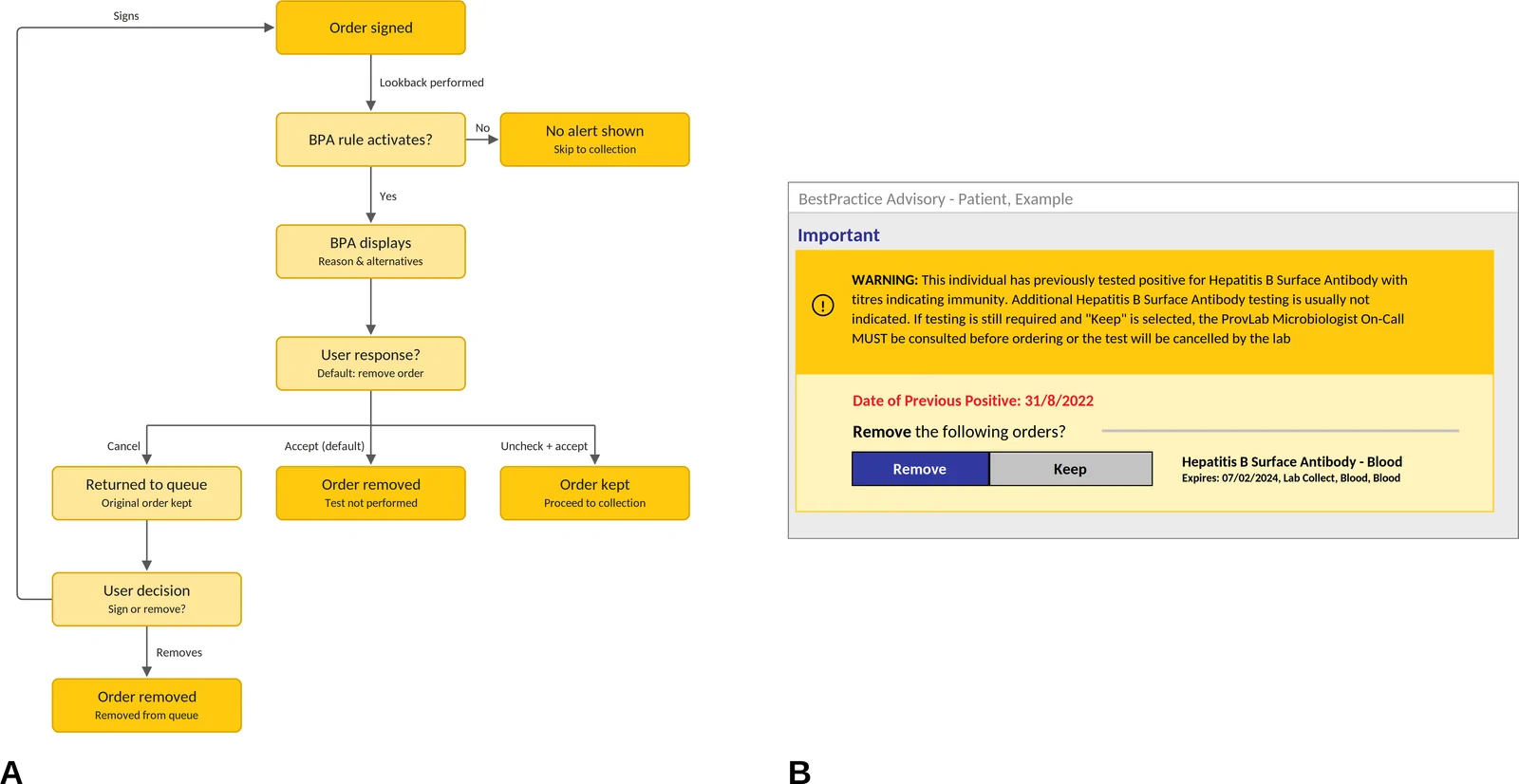
(**A**) Workflow flowchart of the BPA logic from order entry to final decision. (**B**) Example of a hepatitis B virus surface antibody (HBsAb) BPA triggered due to previous positive serology at the point of ordering. Clinicians can override the BPA if necessary.

BPAs were designed by medical microbiologists and reviewed by a clinical decision support committee comprising physician representatives from various disciplines. The BPAs provided real-time decision support when clinicians or laboratory staff ordered infectious disease serology tests, flagging inappropriate tests based on criteria such as historical positivity for IgG antibodies, duplicate test orders within a specified time frame, or a missing prerequisite serology result (Table 1). In cases of a previous positive or duplicate test, the date of the previous test was provided for clinician reference (Fig. 1B). The duplicate-order window (3 days for IgM and 7 days for IgG) was a conservative estimate of the minimum time for an antibody response to develop, meant to catch orders unlikely to reflect a new result rather than define a clinically appropriate retesting interval. The BPAs alerted clinicians using the EHS at the point of order entry, providing patient-specific rationale for why the test is unnecessary and recommending alternatives when appropriate (Fig. 1).

For the auto-cancellation rules, when a test is deemed unnecessary by a similar set of criteria, the system cancels the test after the specimen is entered into the LIS and generates a report documenting the reason for cancellation.

### Data collection

Anonymized data were extracted from the EHS by the Alberta Precision Laboratories (APL) data analytics team beginning 5 months after implementation. Data points included the frequency of BPA activations, criteria that triggered activation, and the number of test cancellations or alternate tests ordered within 24 h following the BPA displays. For auto-cancellation rules, this included the number of tests auto-canceled upon sample receipt in the laboratory and the indication for the auto-cancellation.

### Data analysis

Data were analyzed using descriptive statistics to summarize the outcomes of the intervention. The total number of BPA triggers and their reasons were quantified, as well as the number of tests canceled or modified following BPA interventions. The number of tests auto-canceled by the laboratory auto-cancellation rules was similarly analyzed. We hypothesized that BPAs would be educational and that as more clinicians were exposed, the activation rates would decrease over time. Conversely, increasing rates of user overrides could act as a marker for “alert fatigue” (13, 14). Linear regression (Stata 17.0, StataCorp, College Station, TX) was used to assess trends over time using a significance level of *P* ≤ 0.05 with a Bonferroni correction.

Cost savings were calculated by multiplying the historical reagent cost of each test by the number of cancellations for that specific test and summing across all tests. Cost per individual test was not provided due to organizational policies and agreements with reagent suppliers. The testing platforms used for each test are provided in Table S1. Personnel, overhead, and downstream cascade testing costs were not included. Transportation costs were also not included, as specimens are transported with other tests and other patient samples from collection sites to the laboratory, making it impractical to attribute transportation costs to individual canceled tests.

For venipuncture estimates, we identified all prevented tests that would have required a blood draw and classified whether they were ordered alone or concurrently with other blood tests. Only stand-alone orders were counted as preventing an additional venipuncture. Prevented venipuncture complications were estimated by multiplying the number of venipunctures prevented by a previously published rate of 3.4% (15).

Environmental impact was estimated by extrapolating carbon footprint savings using coefficients from a recent Canadian assessment that applied PAS 2050 methodology (16). Specifically, the carbon intensity per diagnostic test and per phlebotomy procedure was multiplied by the total number of canceled tests, using estimates of 32 g carbon dioxide emissions (CO_2_e) per gold-top tube and 150 g CO_2_e per phlebotomy procedure.

## RESULTS

### BPA activations

Over the 1-year study period, 1,696,709 targeted tests were ordered or attempted to be ordered in the EHS (Table 2). Of these targeted tests, 23,053 (1.4%) resulted in the activation of a BPA, of which 146 (0.6%) rejected the BPA and the testing was ordered, while 22,907 (99.4%) accepted the BPA with no testing ordered. Overall, 12.4% of tests were collected during an inpatient encounter and 87.6% during outpatient encounters.

**TABLE 2:** Individual test list stratified by total tests ordered, performed, and canceled by each intervention

Test	Total tests ordered	BPA alerts	Test avoided by a BPA (%)	User overrode BPA (%)	Alternate test ordered	Auto-canceled (%)	Test performed
Serology							
CMV IgM	6,006	259	252 (4.2)	7 (2.7)		304 (5.3)	5,450
HAV IgG	53,723	850	846 (1.6)	4 (0.5)		6,551 (12.4)	46,326
HAV IgM	28,664	431	426 (1.5)	5 (1.2)		3,229 (11.4)	25,009
HBcAb IgM	5,863	124	116 (2.0)	8 (6.5)		264 (4.6)	5,483
HBcAb total (IgM + IgG)	47,449	686	683 (1.4)	3 (0.4)		1,121 (2.4)	45,645
HBQsAg	4,502	594	594 (13.2)	0			3,908
HBsAb	182,850	5,795	5,692 (3.1)	103 (1.8)		763 (0.4)	176,395
HBsAg	215,792	664	664 (0.3)	0		539 (0.3)	214,589
HCV Ab	250,969	1,296	1,285 (0.5)	11 (0.8)	26	1,880 (0.8)	247,804
HIV-1/2 Ab/Ag	305,597	114	114 (0.0)	0	2	144 (0.0)	305,339
HTLV-1/2 Ab	6,353	3	3 (0.0)	0		13 (0.2)	6,337
Measles IgG	10,914	197	196 (1.8)	1 (0.5)		498 (4.6)	10,220
Mumps IgG	5,414	104	102 (1.9)	2 (1.9)		208 (3.9)	5,104
Prenatal panel^*a*^	58,257	No BPA				Components assessed individually	
Prenatal HIV	58,257	No BPA				18 (0.0)	58,239
Prenatal rubella	58,257	No BPA				25,597 (43.9)	32,660
Prenatal syphilis	58,257	No BPA				539 (0.9)	57,718
Prenatal VZV	58,257	No BPA				26,845 (46.1)	31,412
Rapid syphilis/HIV	1,320	No BPA				14 (1.1)	1,306
Rapid HIV	831	38	38 (4.6)	0		4 (0.5)	789
Rubella IgG	22,632	817	817 (3.6)	0		4,185 (19.2)	17,630
SART panel	1,141	No BPA				123 (10.8)	1,018
Syphilis EIA	283,953	9,599	9,599 (3.4)	0	404	13,681 (5.0)	260,167
VZV IgG	29,708	1,482	1,480 (5.0)	2 (0.1)		3,723 (13.2)	24,505
Total	1,696,709	23,053	22,907 (1.4)	146 (0.6)	432	90,749 (5.3)	1,583,053

^*a*^ Prenatal panels were subjected to rules based on individual components, which include HIV serology, rubella IgG, syphilis screen, and VZV IgG.

The most frequent reason for BPA activation was previously positive serology (19,030, 82.5% of cases), followed by repeat testing performed within 7 (or 3) days (3,429, 14.9%), and a missing prerequisite test (594, 2.6%). BPAs resulted in the ordering of an alternate test on 432 occasions: 404 rapid plasma reagin tests (RPRs) instead of syphilis enzyme immunoassay (EIA) (93.5%), 26 hepatitis C virus (HCV) nucleic acid tests (NATs) instead of HCV serology (6.0%), and 2 HIV NATs instead of HIV antibody/antigen tests (0.5%). Of the 146 tests that were ordered despite the BPAs, 103 (70.5%) were for hepatitis B surface antibody (HBsAb).

### Auto-cancellation rule activation

Of the remaining collected tests, lab auto-cancellation rules canceled 90,749 (5.3%), leaving 1,583,053 of the targeted tests performed during the observational period. The majority of auto-cancellation rules were activated due to previously positive serology (87,884, 96.7%), the same test performed within 7 (or 3) days (2,680, 2.9%), and for unknown reasons (185, 0.2%).

### Most effective BPAs and auto-cancellation rules

The greatest cancellation rates for individual tests due to BPAs were hepatitis B quantitative surface antigen (HBQsAg) serology (13.2% of all orders avoided), VZV IgG (5.0%), and rapid HIV (4.6%). The largest absolute decreases in test volumes attributed to BPAs were for syphilis EIA (9,599), HBsAb (5,692), and VZV IgG (1,480). For auto-cancellation rules, the greatest relative decreases occurred in rubella IgG (36.8% [19.2% prenatal and 43.9% standard]) and VZV IgG (34.8% [13.2% standard and 46.1% prenatal]), while the greatest absolute decreases were seen in VZV IgG (55,917 [24,505 standard and 31,842 prenatal]), rubella IgG (50,290 [17,630 standard and 25,597 prenatal]), and syphilis EIA (13,681 [13,142 standard and 539 pre-natal]).

### Trends over time

The BPA and auto-cancellation rule trends were stable for most tests, except for those in Fig. S1. The proportion of tests resulting in BPAs for syphilis serology and HBsAb increased by 0.23%/month ([0.11 to 0.36], *P* = 0.002) and 0.09%/month ([0.06 to 0.12], *P* < 0.0001), respectively. For auto-cancellation rules, the proportion of canceled HBsAb increased by 0.02%/month ([0.01 to 0.03], *P* = 0.0002). In May 2024, the provincial sexually transmitted infection (STI) clinics began using the EHS, corresponding to a rise in syphilis BPA firing.

### Savings and sustainability

Over the 1-year period, BPAs and auto-cancellation rules saved a combined $260,138 CAD ($192,502 USD) in laboratory reagents alone (BPAs saved $52,049 CAD [$38,516 USD] and auto-cancellation $208,089 CAD [$153,986 USD]). For BPAs, the tests with the greatest savings were HBsAb, syphilis EIA, and hepatitis A (HAV) IgG, while prenatal panels, HAV IgG, and syphilis EIA showed the highest cost savings for auto-cancellations. Fig. S2 shows the number of blood draws that were prevented through BPAs. Using the historical complication rate of 3.4%, BPAs potentially prevented 569 complications associated with venipuncture (15). Regarding environmental impact, BPAs saved 2,512 kg CO_2_e (carbon dioxide equivalent) by preventing phlebotomies and an additional 690 kg of CO_2_e through reduced use of gold-top blood vials (16).

## DISCUSSION

Our findings demonstrate that both BPAs and auto-cancellation rules were effective in decreasing a wide range of unnecessary infectious disease serology tests, with a combined 6.7% of the targeted tests prevented, reflecting 113,656 tests (Table 2). This aligns with previous studies showing that diagnostic decision support can substantially curb redundant lab investigations (6, 10, 11, 17). Duplicate orders were the most common reason for cancellation in our study, consistent with other studies such as Graham et al. (10), whose alerts specifically targeted duplicate ordering across six tests (urine culture, fecal PCR, hepatitis B and C PCR, and EBV and CMV serology) and achieved substantial reductions, further supporting duplicate detection as a high-yield target for diagnostic stewardship interventions (10).

Auto-cancellation rules were more effective than BPAs (5.3% vs 1.4% of orders, respectively), likely because of the higher volume of tests ordered on paper requisitions (instead of the EHS) by clinicians in outpatient community practices. Consistent with this observation, specimens were collected approximately sevenfold more frequently in outpatient than inpatient settings. However, collection setting is an imperfect proxy for whether orders were subject to BPAs, as some sites providing collection services for community patients are classified as inpatient encounters in the provincial EHS, and the denominator of paper-based orders eligible only for auto-cancellation was not available. Theoretically, with the implementation of a universal EHS, there would be no need for auto-cancellation rules, except in cases where a clinician has overridden a BPA erroneously.

With EHS alerts, there is always the concern of “alert fatigue” (13). Ancker et al. (13) followed six BPAs over time and found that the acceptance rate gradually decreased for three of them over a 3-year period. Encouragingly, throughout our study, we did not observe an erosion of effectiveness, providing no evidence of alert fatigue. Adherence to the BPAs was high, with 99.4% resulting in a canceled test, though because adherence reflects the downstream action taken rather than clinician engagement with the alert content itself, we cannot fully exclude a milder form of fatigue in which the alert was passively accepted rather than actively reviewed. This suggests that our BPA design with patient-specific information and targeted orders that were clearly duplicative or contraindicated may have helped reduce alert fatigue. It is possible that we could see a change beyond the study period.

Of the overridden BPAs, the majority were for HBsAb. Niès et al. (6) similarly found that HBV testing was frequently ordered despite alerts. Their 24% compliance rate with the HBsAb alert was lower than ours (98.2%). This difference may be explained because our BPA was activated when there was a previous result with protective titers (≥ 10 IU/L) or a repeat within 1 week, whereas the previous study activated with previous negative titers. This highlights the importance of clinically relevant alert rule selection. Our study lacked data on why BPAs were overridden. Plausible explanations include misinterpretation of prior serologic results, picking the first test that appears in order lists, need to document immunization status for occupational health reasons, default orders embedded in order sets, added to personal preference lists, or institutional policies requiring the test for administrative purposes (18, 19). This underscores the value of involving stakeholders such as laboratory medicine, infectious diseases, obstetrics/prenatal programs, transplant programs, public health, legal/compliance, and clinical informatics in BPA design, which can help ensure that rules remain clinically appropriate and locally compliant.

We expected the BPA and auto-cancellation comments to educate clinicians and subsequently lead to decreased activations over time. Contrary to our hypothesis, activations for most tests plateaued during the study, whereas syphilis EIA and HBsAb increased (Fig. S2). Testing does not occur in a vacuum, and the increase in syphilis EIA BPA activation may have been in response to rising syphilis rates in the province and the STI clinics going live with the EHS at the study midpoint (20). Similar to our findings, Niès et al. (6) implemented an automated alert for HBsAg and found that the previously increasing rate of redundant tests plateaued and remained stable over time (6). We are unaware of any specific concurrent educational interventions during our study period, but tracking BPA firing rates going forward could serve as a useful metric for institutions to monitor the effectiveness of future targeted education efforts.

### Financial, environmental, and patient benefits

These interventions yielded substantial benefits and saved approximately $260,000 CAD ($192,000 USD) in reagent costs. Since some alerts were not active for the full study, annual savings are likely higher. Other studies have had similar financial success in reducing chemistry/hematology tests (11, 21), microbiologic cultures (22, 23), and serologic investigations (10). To the best of our knowledge, our study is the largest non-culture-based microbiology-focused intervention.

Furthermore, these savings represent only reagents and do not include costs associated with labor, collection devices, phlebotomy, the review of inappropriately ordered tests by laboratory medical or technical staff, lower downstream diagnostic work (i.e., following up on false positives), and longer patient stays resulting from delayed diagnosis.

Beyond financial savings, this reduction in unnecessary testing would also reduce false-positive results and their downstream consequences, including patient stress, repeat testing, and specialist referrals. These cascading effects extend beyond the individual patient, contributing to inefficient use of clinician time and healthcare resources more broadly.

Venipuncture, while a relatively safe medical procedure, comes with risks, both physical and emotional (15, 24). We estimated that BPAs prevented 569 complications associated with phlebotomy. Fewer blood draws and laboratory tests also improve environmental sustainability. Quantifying environmental impact is increasingly relevant to the field of diagnostic stewardship, as healthcare systems face growing pressure to reduce their carbon footprint alongside clinical and financial goals (25). Reporting the estimated CO_2_e savings associated with reduced unnecessary testing provides institutions with a metric to communicate the environmental value of stewardship interventions, which may help justify the upfront resource investment required to design and implement BPAs and auto-cancellation rules. Graham et al. (10) found that by canceling 200 CMV and EBV serologies, they could prevent 17 kg CO_2_e (10), and a Canadian study estimated that the emissions associated with drawing blood into a serum separator tube were 182 g of CO_2_e per draw (16). Assuming similar environmental costs, our BPAs saved 3,201 kg CO_2_e, a significant amount, equivalent to driving a gasoline-powered car 13,117 km (26).

### Limitations

Our study has several limitations. As a retrospective, non-qualitative study, we did not prospectively collect detailed reasons for overrides, clinician feedback, or impact on patients. Capturing this information in future work could help refine BPA logic and better target alerts. We also did not determine individual clinician ordering to identify whether specific clinicians contributed more than others to the outcomes. Due to multiple changes in lab organization, testing processes, testing locations, and LISs, we could not assess the ordering practices pre-intervention. Furthermore, indications and protocols for tests can change, as do epidemiologic factors (i.e., increased syphilis rates in our setting [20]), leading to further variability. As this was a pilot implementation, the intervals used to define duplicate testing (3 and 7 days) were deliberately conservative. Expanding these windows (e.g., 2–4 weeks for IgM and 1–2 months for IgG) may increase the impact of these interventions. Finally, our setting is a large, integrated provincial health system with centralized labs, which may limit generalizability but highlights the benefits to an integrated health system and accompanying EHS.

### Conclusions

Both BPAs and auto-cancellation rules effectively decrease unnecessary serologic tests. BPAs did not appear to be associated with alert fatigue and provided patient-specific information to inform clinicians, resulting in 99% adherence. By intercepting orders before specimen collection, BPAs offer additional cost savings and spare patients unnecessary procedures compared to auto-cancellation rules, which act only after the sample has been collected. Since BPAs cannot catch orders placed outside the EMR, combining both interventions is the best approach for maximizing coverage and impact. Development of BPAs for other infectious disease tests such as nucleic acid tests could further improve patient care.

## Data Availability

Requests for data may be made to the corresponding author.

## SUPPLEMENTAL MATERIAL

Supplemental material jcm.00770-26-s0001.docx Fig. S1 and S2; Table S1.

ASM does not own the copyrights to Supplemental Material that may be linked to, or accessed through, an article. The authors have granted ASM a non-exclusive, world-wide license to publish the Supplemental Material files. Please contact the corresponding author directly for reuse.
